# Eye to Eye in the Village

**DOI:** 10.3201/eid1412.000000

**Published:** 2008-12

**Authors:** Polyxeni Potter

**Affiliations:** Centers for Disease Control and Prevention, Atlanta, Georgia, USA

**Keywords:** Art-science connection, emerging infectious diseases, art and medicine, Marc Chagall, zoonoses, human-animal diseases, one medicine, modern art, cubism, I and the Village, about the cover

**Figure:**
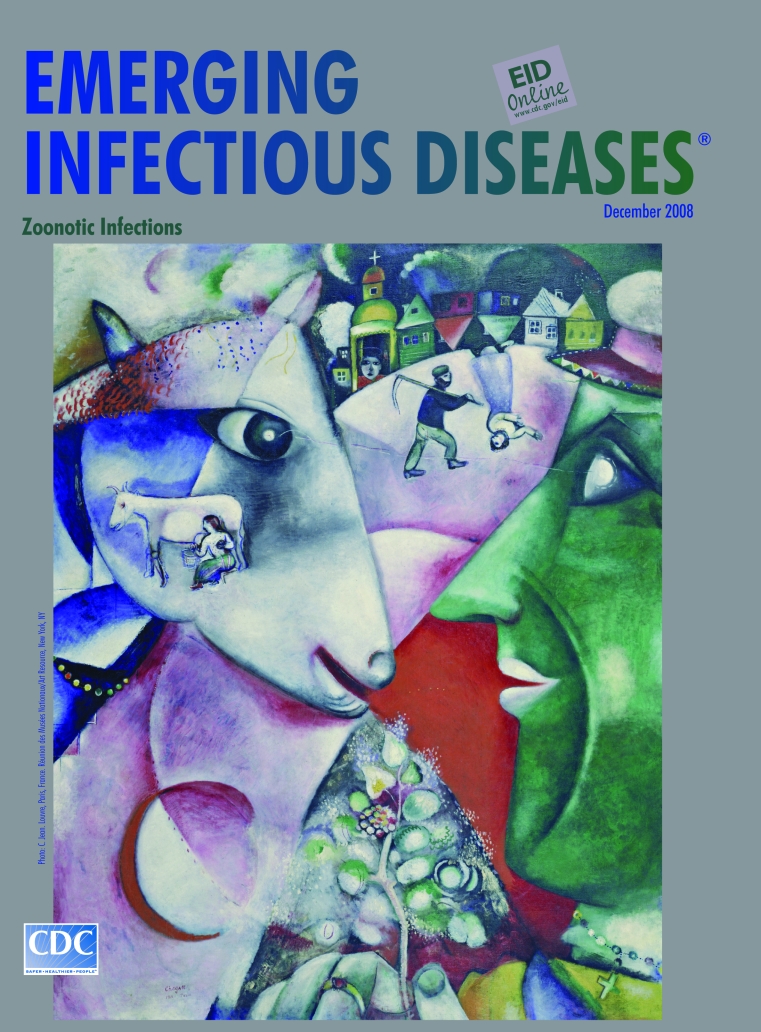
**Marc Chagall (1887–1985) I and the Village (1911)** Oil on canvas (192.1 cm × 151.4 cm) Copyright ARS, NY. Mrs. Simon Guggenheim Fund (146.1945) Digital Image Copyright the Museum of Modern Art/Licensed by SCALA/Art Resource, NY. The Museum of Modern Art, New York, NY, USA

“One fine day as my mother was putting the bread in the oven,” recounted Marc Chagall, “I went up to her and taking her by her flour-smeared elbow I said to her, ‘Mama I want to be a painter.’” This wish was out of line with the circumstances. The oldest of nine children in a family of modest means, he was aware of the constraints and his father’s toil in the fish-curing business, “My heart used to twist like a Turkish bagel as I watched him lift those weights and stir the herring with his frozen hands.”

Chagall had little exposure to art during childhood, until one day, he saw a schoolmate draw a picture from a magazine. Ridiculed for his astonishment, he revolted, “It roused a hyena in me.” He started copying from magazines himself and showed early talent. Later, Russian icons, which he found “magical and unreal,” also provided inspiration. The family lived in Vitebsk, Byelorussia (now Belarus), then part of the Russian Empire. This small village community fueled his life’s work with honesty and charm, poetry and humor, lyricism and pure joy.

Chagall’s studies began in 1906 under local artist Yehuda Pen. “I learned about Pen when I was riding on a tram. It was crossing the Cathedral Square and I saw a signboard―white letters on blue: ‘Artist Pen’s School.’ ‘What a cultured city is our Vitebsk’ I thought.” Soon he moved to St. Petersburg to join the Society of Art Supporters School, where he studied briefly under Nikolas Roerich. At the time, Jewish residents needed a permit to live in the city, so he was briefly jailed for violating this restriction. He continued under Léon Bakst at the Zvantseva School of Drawing and Painting.

His reputation as an artist grew solid enough to earn him sponsorship to Paris in 1910, the heyday of modern art. Fauvism, with its emphasis on emotion and color, was still influential, and cubism, with its turn to representation from multiple points of view, was bursting onto the scene, led by Picasso and Braque. “I aspired to see with my own eyes what I had heard of from so far away …. The sun of Art then shone only on Paris.”

He settled in the heart of the arts community, Montparnasse, and met avant-garde poets Blaise Cendrars and Guillaume Apollinaire and painters Robert Delaunay and Fernand Léger. He studied broadly and produced during these years some of his most famous village paintings. “In Paris, it seems to me I have found everything, but above all, the art of craftsmanship. I owe all that I have achieved to Paris, to France, whose nature, men, the very air, were the true school of my life and art.”

His studio was in La Ruche (The Beehive), an artists’ colony. Amedeo Modigliani lived on the same floor. “I used to stand alone … in front of my easel in the wretched light of a paraffin lamp …. It was between those four walls that I wiped the dew from my eyes and became a painter.” The fauvists freed his use of color, and he learned from the cubists, though he never joined them: “Let them eat their fill of their square peas on their triangular tables.” Cendrars, who titled some of his paintings, wrote his own poetic portrait of Chagall: “He’s asleep/He’s awake/Right away he’s painting/He grabs a church and paints with the church/He grabs a cow and paints with the cow/With a sardine/With heads, hands, knives/He paints with an oxtail/….”

Soon he was exhibiting at the Salon des Indépendants, and though his works were not selling well at this the height of abstract art, he espoused neither impressionism nor cubism. Instead, he used their influences to articulate his own unique style, a blend of formal structure, brilliant color, and fluid form. “For the cubists,” he wrote, “a painting was a surface covered with forms in a certain order. For me a painting is a surface covered with representations of things―objects, animals, human beings―in a certain order in which logic and illustration have no importance.”

When Apollinaire saw the paintings at Chagall’s studio in Montparnasse, he pronounced them *surnaturel* (supernatural), perhaps seeing in them the first signs of surrealism before he even coined the term. A poem he wrote about Chagall whom he mysteriously dubbed “Rotsoge,” captured the painter’s conflicted spirit―now avant-garde Paris, now the village: “Your scarlet face your biplane convertible into hydroplane/Your round house where a smoked herring swims.”

The whimsy, unreality, and sheer exuberance of his compositions would later attract the attention of surrealists, who enthusiastically claimed him as one of their own. Despite his influence on them, however, he did not share their inspiration from the subconscious, the world of dreams. He drew from his experiences, “You see, here is my whole biography: They used to find my grandpa on the roof, he loved eating *tzimmes* there. And my uncle loved walking the street with just a nightshirt to his body.” When at age 35 he finished writing his autobiography, My Life, it seemed he wrote it to explain his work.

After a very successful art show in 1914, Chagall went back to Vitebsk for a brief visit, which ended up lasting several years on account of World War I. In Vitebsk, he embraced the Bolshevik Revolution and was made commissioner of fine arts by the new Soviet government. He organized exhibitions and became Director of the Vitebsk Academy of Art. But with time, it became clear that neither his style nor his vision for society fit in. He moved to Moscow, where he painted theater murals and designed costumes and sets until he left Russia in 1922.

Back in Paris André Breton offered him the approval of surrealists, “With Chagall alone, the metaphor made its triumphant return into modern painting.” But he resisted, “I want an art of the earth and not merely an art of the head.” He followed his own style, traveled widely, and ventured into multiple media (ceramics, mosaics, stained glass). World War II forced him to flee France, this time to New York City. Three years after the end of the war, he returned to France, where he lived the rest of his life. His art career spanned more than 70 years. He left behind thousands of works, a legend in his own time.

Painted a year after Chagall first went to Paris, I and the Village, on this month’s cover, integrates elements of cubism with his own vision of the world, “Lines, angles, triangles, squares, carried me far away to enchanting horizons.” A geometric structure frames a village scene with overlapping images: a green-faced man with a cap, his hand holding a flowering sprig, stares directly at a cow’s head. The cow stares back. The sun, the earth, and the moon in eclipse blend softly in the foreground. A peasant carries a scythe. A female fiddler floats upside down along with two houses from the row above. The village skyline crowns the horizon.

“Cows, milkmaids, rooster, and provincial Russian architecture … are part of the environment from which I spring,” Chagall wrote, but individual parts assume roles and dimensions he created for them. “I fill up the empty space in my canvas as the structure of my picture requires with a body or an object according to my humor.” He explains, “In the large cow’s head…I made a small cow and woman milking visible through its muzzle because I needed that sort of form, there, for my composition.”

I and the Village seems Chagall’s personal notion of the community, a cosmic one (humans, animals, habitat, stars), made intimate and poetic, jewel-colored, folksy, with music, sparkle, and humor. Two main characters, man and beast stand face to face at the entrance, deliberately linked with a line, eye to eye.

Barnyard animals, cows or goats, frequent Chagall’s work, wandering freely, often airborne along with humans, unconstrained by reality. Here, they share equivalent status across the planets in the entrance of the village. In a playful standoff, each claims the “I,” seeing alternatingly fences and tethers, limits and yoke; milk and honey, highways and byways. Though wide-eyed, they seem oblivious to what weighs all of us down to earth: infection and consequent illness and death, shared lavishly in the village. From the narrow perspective of “us” or “them,” zoonotic infections are not well understood. Their solution requires the total picture, from the human and the animal point of view. Because each “I” sees a different village.
